# Genetic analyses of longitudinal phenotype data: a comparison of univariate methods and a multivariate approach

**DOI:** 10.1186/1471-2156-4-S1-S29

**Published:** 2003-12-31

**Authors:** Qiong Yang, Irmarie Chazaro, Jing Cui, Chao-Yu Guo, Serkalem Demissie, Martin Larson, Larry D Atwood, L Adrienne Cupples, Anita L DeStefano

**Affiliations:** 1 Departments of Biostatistics, Boston University, Boston, Massachusetts, USA; 2 Departments of Neurology, Boston University, Boston, Massachusetts, USA; 3 Departments of Mathematics and Statistics, Boston University, Boston, Massachusetts, USA; 4 Departments of Medicine, Boston University, Boston, Massachusetts, USA

## Abstract

**Background:**

We explored three approaches to heritability and linkage analyses of longitudinal total cholesterol levels (CHOL) in the Genetic Analysis Workshop 13 simulated data without knowing the answers. The first two were univariate approaches and used 1) baseline measure at exam one or 2) summary measures such as mean and slope from multiple exams. The third method was a multivariate approach that directly models multiple measurements on a subject. A variance components model (SOLAR) was employed in the univariate approaches. A mixed regression model with polynomials was employed in the multivariate approach and implemented in SAS/IML.

**Results:**

Using the baseline measure at exam 1, we detected all baseline or slope genes contributing a substantial amount (0.08) of variance (LOD > 3). Compared to the baseline measure, the mean measures yielded slightly higher LOD at the slope genes, and a lower LOD at the baseline genes. The slope measure produced a somewhat lower LOD for the slope gene than did the mean measure. Descriptive information on the pattern of changes in gene effects with age was estimated for three linked loci by the third approach.

**Conclusion:**

We found simple univariate methods may be effective to detect genes affecting longitudinal phenotypes but may not fully reveal temporal trends in gene effects. The relative efficiency of the univariate methods to detect genes depends heavily on the underlying model. Compared with the univariate approaches, the multivariate approach provided more information on temporal trends in gene effects at the cost of more complicated modelling and more intense computations.

## Background

In genetic studies, subjects may be measured repeatedly over a period of time to monitor how the quantitative traits change with age (or other time measure). These types of data offer great opportunity to evaluate whether a gene's influence on traits changes with age. Univariate variance components approaches that use a single measurement or summary statistics such as mean and slopes are easy to implement and the results have a straightforward interpretation. However, the univariate approaches may not be extracting the full information content of the data and may not provide information about differing genetic effects with age. Multivariate variance components approaches that directly model all measurements on one subject by estimating covariance structures within or between subjects may better utilize the information in the data set and provide age-specific estimates of genetic effects at the cost of greater computational burden and more complex interpretation of the linkage information.

In this work, we compared three approaches (two univariate and one multivariate) to analyze repeated measures in genetic studies. The first two approaches used univariate phenotypes that were either based on a single exam measurement or summaries from multiple exam measurements. Variance components models for univariate phenotypes were applied [1]. The third method used multiple available measurements on each subject as a multivariate phenotype. We modelled the random genetic and subject-specific random environmental effects as orthogonal polynomials of age in a mixed regression model and implemented it in SAS/IML.

We applied the three approaches to analyze total cholesterol levels (CHOL) in replicate 8 of the Genetic Analysis Workshop 13 simulated data without prior knowledge of the answers.

## Methods

### Univariate Approaches

#### Baseline Measure

Baseline measure of CHOL at Exam 1 of both cohorts was used as the dependent variable in variance components model analyses implemented in SOLAR [1]. Total heritability (h^2^) was estimated as the proportion of the total phenotypic variance due to the additive polygenic variance. SOLAR calculates a LOD score by taking log_10 _of the ratio of the maximum likelihood of a linkage model (containing a quantitative trait loci (QTL) variance and a residual polygenic variance component) to that of a purely polygenic model. The QTL h^2 ^was computed as the proportion of the QTL variance to the total phenotypic variance. In multipoint analyses, linkage to adjacent markers was also considered to evaluate the linkage to the current marker using a regression approach [1]. Covariates including gender, age, systolic blood pressure, and height were adjusted for in regression models prior to the heritability and linkage analyses.

#### Summary Measures

In calculating summary measures of the repeated CHOL measurements, we looked at three definitions of the mean by imposing restrictions on the selection of the subjects and their measurements. Definition 1 (D1) required that subjects had CHOL measured for at least three exams. This definition resulted in subjects with a wide range of observations used, from 3 to 15. We were concerned that the different number of exams, and hence different standard error associated with the mean measure, would affect the genetic analysis and explored definitions in which each summary measure was based on a similar number of exams. To obtain, approximately, an equal number of exams for both cohorts, definition 2 (D2) included only the first five exams of both cohorts, and all subjects had to have CHOL measured for at least two exams. For D2, Cohort 1 and 2 members had measures taken at approximately the same age (45 years). To obtain measures taken at approximately the same chronological time in the two cohorts, definition 3 (D3) included only exams 10, 14, 15, and 20 for Cohort 1 and exams 1–5 for Cohort 2, and required all subjects have CHOL measured for at least two exams. A slope of CHOL versus age was computed for each individual satisfying D1. Heritability and linkage analyses were conducted in the same way as for the baseline measure.

### Multivariate Approach

We set up a mixed regression models as follows

*y*_*ij *_= *X*_*ij *_β + *g*_*ij *_+ *r*_*ij *_+ ε_*ij*_,

where *y*_*ij *_is the CHOL at the age *j *for subject *i*, *X*_*ij *_and β are vectors of covariates and coefficients of fixed effects, *g*_*ij *_and *r*_*ij *_are subject-specific additive genetic and environmental effects (i.e., repeated measurement effects) respectively, and ε_*ij *_is the residual environmental effect of subject *i*. To allow age-varying effects, *g *and *r *are modelled by Legendre polynomials similar to the approach in Meyer [2]:



where {α_*im *_| *m *= 0, ..., *k*_*A *_- 1} ~ *N*(**0**, ∑_α_) and {γ_*im *_| *m *= 0, ..., *k*_*R *_- 1} ~ *N*(**0**, ∑_γ_) are random regression coefficients of additive genetic and environmental effects for subjects *i*, φ_*m *_() is the *m*^th ^Legendre polynomial [3] evaluated at  (which is age *j *standardized to the interval [-1,1] by the age range observed in the data), *k*_*A *_and *k*_*R *_are the order of the corresponding polynomials. The covariance between two observations of two subjects is then equal to equation (1), assuming *g *and *r *independent of each other,



It can be further simplified by assuming *Cov*(α_*im*_), α_*i'l *_= 2Φ_*ii' *_*Cov*(α_*m*_), α_*l*_) and *Cov*(γ_*im*_, γ_*i'l )*_= 2δ_*ii' *_*Cov*(γ_*m*_, γ_*l*_), where Φ_*ii*_, is the kinship coefficient, δ_*ii*' _= 1, if *i *= *i*' and 0 otherwise, and , if *i *= *i*' and *j *= *j*', and 0 otherwise. The total h^2 ^at a standardized age t* is therefore



We extended the model to incorporate the effect of a QTL by adding a Legendre polynomial with random coefficients η_*m*_, *m *= 1, ..., *k*_*Q*_, ~ *N*(0, ∑_η_). The covariance contribution from this QTL to equation (1), assuming its independence of *g *and *r*, is , where π_*ii' *_is the multipoint shared by the two subjects at the QTL. Then the QTL h^2 ^due to this locus is



We utilized kinship coefficients and multipoint identity by descent (IBD) computed in SOLAR and read these values into a matrix using SAS/IML. The other parameters



were estimated via a nonlinear maximization procedure NLPQN in SAS/IML [4].

Since computational load increased quickly with the number of observed ages, we divided the 70 distinct ages (ranging from 20 to 93) into five intervals: below age 30, with 10-year increments from age 30 to 60 and greater than 60. Order of polynomials was set as 2 (i.e., *k*_*A *_= *k*_*R *_= *k*_*Q *_= 3) for both polygenic and subject-specific environmental effects and 1 for QTL effects. For those individuals who had more than one exam in an age interval, the average phenotype and covariates measured during that age interval were used in the analyses. Since it was time consuming to carry out genome-wide analyses, we only implemented this analysis at the three linked loci (S7, B30, B32) found in the univariate analyses.

## Results

### Univariate Approaches

We compared our results to the simulating model in Table 1. Since there was no substantial difference in heritability or multipoint LOD scores between the three definitions of means, we only presented the results for mean D2. The total h^2 ^of baseline, mean D2, and slope measures were estimated as 0.55, 0.60, and 0.42, respectively. Using the baseline measure, we detected (LOD > 3.0) one of the three slope genes, S7 (QTL h^2 ^= 0.20), and three of the four baseline genes, B30 (QTL h^2 ^= 0.27), B31 (QTL h^2 ^= 0.21), and B32 (QTL h^2 ^= 0.30). Using mean measure D2, we were able to detect the slope gene S7 (QTL h^2 ^= 0.33) and the baseline gene B32 (QTL h^2 ^= 0.26). Using the slope measure, only slope gene S7 (QTL h^2 ^= 0.33) was detected. There were one, two, and one false positives for the Exam 1, mean D2, and slope measures, respectively, and the LOD scores of the false positives were between 3.6 and 4.3 (Table 1).

### Multivariate Approach

**Figure 1:**
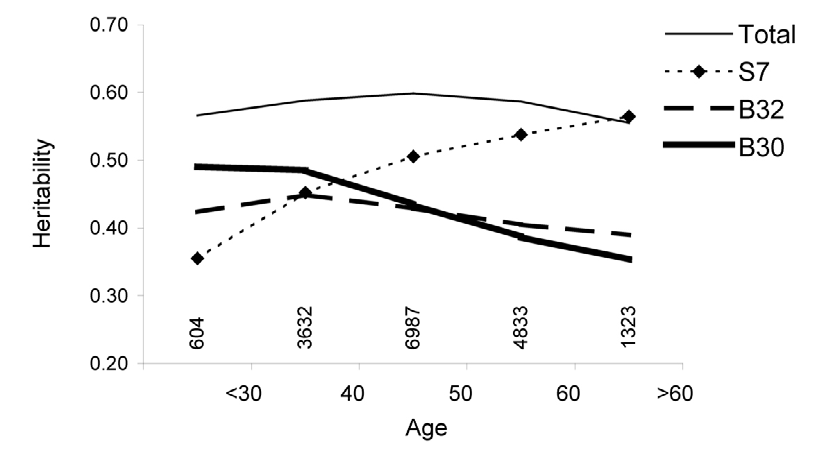
**Total and QTL Heritabilities **The total and QTL heritability curves against age for S7, B32, and B30. The numbers above the x-axis are the number of observations in each age interval.

Among the 2701 subjects who had at least one measurement of CHOL, there were 70, 670, 1950, and 10 subjects who had one to four repeated measurements respectively, taken over the five age intervals. The estimated total h^2 ^was 0.57, 0.59, 0.60, 0.59, and 0.55 in the five age groups. The QTL h^2 ^for S7 ranged from 0.35 to 0.56. The QTL h^2 ^for B30 and B32 ranged from 0.39 to 0.45 and 0.35 to 0.49, respectively. The total and QTL h^2 ^estimates were presented in Figure 1. The total h^2 ^and the QTL h^2 ^curves of B30 and B32 were relatively flat and slightly declining with age. The slope gene, S7, had a monotonic increase in its QTL h^2 ^with age.

## Discussion

We have presented two univariate and one multivariate approach to analyze longitudinal phenotype data. The univariate approaches were successful in identifying genes for this generating model. The multivariate approach provided additional descriptive information on changes in gene effects with age.

We found the relative efficiency in the first two approaches (baseline or summary measures) depended heavily on the generating model. Since CHOL were generated using a basic linear model of age (CHOL = Chol_base + Chol_slope * age + random_error), using baseline measure at Exam 1 in which the age of subjects spanned between 20 and 85 enabled us to detect all slope and baseline CHOL genes except three genes with a variance <0.08. The mean measure seemed to contain more noise than Exam 1 data for detecting the baseline genes, but produced a slightly higher LOD than the slope measure to detect slope genes. This observation was confirmed in an experiment: when there is considerable residual random error in the trait, the slope measure could be inferior to the mean measure in power to detect a slope gene [5].

The results of the three definitions of means were not very different for this generating model, though they were designed to avoid possible shortcomings in the other definitions (See Methods section). In practice, one definition may be better than the others depending on the characteristics of the data.

The total h^2 ^estimations from the multivariate approach did not vary much with age and were close to those estimated from the univariate approaches using Exam 1 or mean D2. The QTL h^2 ^for B30 and B32 estimated from multivariate analyses were higher than those obtained from univariate analyses, especially at younger ages. The difference at younger ages may be caused by more aged subjects in Exam 1 and mean D2 measures that resulted in lower proportion of total phenotypic variance (increasing with age) explained by the baseline genes for this generating model. The QTL h^2 ^for slope gene S7 estimated using slope measure was close to that estimated using multivariate measure for those aged 30 or less. In theory, QTL variance for S7 from the multivariate measure should be approximately equal to that from slope measures multiplied by age^2 ^for this generating model, which explains the monotonic increase of QTL h^2 ^for S7 observed from the multivariate approach.

Compared with the univariate approaches, the multivariate approach provided more information regarding the temporal trend of gene effects during aging. We were not able to tell which gene(s) affected the baseline or slope using the univariate approaches, since the univariate measures overlapped with each other in the ability to detect slope and baseline genes. Using the third approach, the QTL h^2 ^for the two baseline genes were nearly flat and slightly declining with age, but that of the slope gene showed a clear trend of monotonic increase with age, which distinguished the slope gene from the baseline genes.

In conclusion, we found univariate approaches were capable of discovering some of the important trait genes with simple modelling and feasible computational load. The multivariate approaches can provide additional information on age-varying effects of genes but generally involves heavy computation and complex modelling. More work is needed to further develop the multivariate approach in areas such as a sensible test of significance. Nevertheless, the multivariate approach shows promise for genetic analyses of longitudinal measures in linkage studies.

## Figures and Tables

**Table 1:** Linkage analyses results for mean, slope and baseline measure at Exam 1

			Multipoint LOD scores at CHCL genes (QTLh^2^)							
				
			Slope Genes			Baseline Genes				
				
	No. Subjects	Total h^2^	S7	S8	S9	B30	B31	B32	B33	Number of False Positives (LOD scores)
Baseline	2869	0.55	**3.1 **(.20)	0.6	0.0	**5.3 **(.27)	**3.1 **(.21)	**8.1 **(.30)	0.0	1 (**3.1**)
Mean	2812	0.60	**10.6 **(.33)	1.3	0.0	2	2.8	**6.8 **(.26)	0.0	1 (**3.3**)
Slope	2698	0.42	**10.3 **(.33)	0.25	0.0	0.0	0.0	0.0	0.0	2 (**4.3, 3.6**)

The table contains number of subjects, total heritability and multipoint LOD scores at slope or baseline CHOL genes; and LOD score peaks ≥ 3 at locations where no CHOL genes existed (false positives) for baseline measure at Exam 1, mean definition 2 and slope. LOD scores in bold indicate genome-wide significant results using LOD = 3 as a threshold. QTL heritabilities for the detected true genes were also presented next to the LOD scores.
